# Rumen and Fecal Microbiota Characteristics of Qinchuan Cattle with Divergent Residual Feed Intake

**DOI:** 10.3390/microorganisms11020358

**Published:** 2023-01-31

**Authors:** Xiaonan Zhou, Ying Ma, Chaoyun Yang, Zhiyan Zhao, Yanling Ding, Yanfeng Zhang, Pengfei Wang, Lei Zhao, Chenglong Li, Zonghua Su, Xiaowei Wang, Wenxun Ming, Ling Zeng, Xiaolong Kang

**Affiliations:** Key Laboratory of Ruminant Molecular and Cellular Breeding, School of Agriculture, Ningxia University, Yinchuan 750021, China

**Keywords:** metagenome, beef cattle, microbiota, rumen and manure, residual feed intake

## Abstract

Residual feed intake (RFI) is one of the indicators of feed efficiency. To investigate the microbial characteristics and differences in the gastrointestinal tract of beef cattle with different RFI, a metagenome methodology was used to explore the characteristics of the rumen and fecal microbiota in 10 Qinchuan cattle (five in each of the extremely high and extremely low RFI groups). The results of taxonomic annotation revealed that *Bacteroidetes* and *Firmicutes* were the most dominant phyla in rumen and feces. *Prevotella* was identified as a potential biomarker in the rumen of the LRFI group by the LEfSe method, while *Turicibacter* and *Prevotella* might be potential biomarkers of the HRFI and LRFI group in feces, respectively. Functional annotation revealed that the microbiota in the rumen of the HRFI group had a greater ability to utilize dietary polysaccharides and dietary protein. Association analysis of rumen microbes (genus level) with host genes revealed that microbiota including *Prevotella*, *Paraprevotella*, *Treponema*, *Oscillibacter*, and *Muribaculum*, were significantly associated with differentially expressed genes regulating RFI. This study discovered variances in the microbial composition of rumen and feces of beef cattle with different RFIs, demonstrating that differences in microbes may play a critical role in regulating the bovine divergent RFI phenotype variations.

## 1. Introduction

Ruminants rely on rumen microbiota (mainly composed of bacteria, archaea, fungi, and protozoa) to promote feed digestion, and provide nutrients such as short-chain fatty acids (SCFAs) and microbial proteins to the host [1], of which the acetic acid, propionic acid, and butyric acid fulfill 70% of the energy requirements of the host organism [1,2]. Residual feed intake (RFI) is the difference between predicted and actual feed intake [3], and is considered a valid indicator of animal feed efficiency [4]. Moreover, animals with low RFI consume >20% less feed than those with high RFI while maintaining the same body weight and tissue growth [5]. Factors such as digestion, metabolism, and physical condition (health) of the host all influence RFI in animals. In addition, gut microbiota plays a crucial role in nutrient metabolism and energy utilization. The energy metabolism of the body is significantly correlated with the level of RFI [4,6], and genes and species of the rumen microbiome could predict variation (91% accuracy) of RFI phenotypes of dairy cattle [6]. The abundance of *Lactobacillus* and *Ruminobacter* has been reported to correlate with RFI in the intestine [7]; meanwhile, lower abundances of *Methanosphaera* stadtmanae and *Methanobrevibacter* sp. are found in animals with high RFI [8]. Furthermore, several microbiotas have heritability, composition and abundance of the microbiota that have a wide specificity and diversity at different growth stages and intestinal sites [9]. In a recent study, the authors mapped a quantitative trait locus affecting the abundance of *Erysipelotrichaceae* species and showed that it was caused by a 2.3 kb deletion in the gene encoding N-acetyl-galactosaminyl-transferase, which decreases the concentrations of N-acetyl-galactosamine in the gut and thereby reduces the abundance of *Erysipelotrichaceae* that can import and catabolize N-acetyl-galactosamine [10]. Currently, it is widely accepted that microbiota affects the metabolic activity and health of the body; it has been revealed that rumen microbes explain approximately 20% of the variation in feed efficiency traits of beef cattle [11,12]. Interestingly, the amplicon sequence variant of several genera (including *Vibrio anaerobic*, *Bacillus mimicus*, *Bacillus cereus*, *Bacillus faecalis*, and *Lactobacillus*) is significantly correlated with feed efficiency, and this effect exists at different growth stages in monogastric animals [13]. Microbes regulate numerous biological functions of the host [14], and numerous studies have shown that microbes regulate fat deposition in animals [15,16]. Thus, animals with different phenotypes may have their specific microbiota characteristics in the gastrointestinal tract at the genetic level.

We hypothesized that the fecal or rumen microbiota profiles differed in divergent RFI beef cattle, which may have an important regulatory effect on the RFI phenotype of beef cattle. In this study, we investigated the rumen and fecal microbiota profiles of Chinese indigenous beef cattle (Qinchuan cattle), to clarify whether specific microbial genera correlate with phenotypic variation in the RFI of the host and whether there are RFI-related dominant genera in order to expand the understanding of microbial regulation of bovine RFI phenotypes and improve cattle feed efficiency through artificial interventions in the future.

## 2. Materials and Methods

### 2.1. Collection of Test Animals and Samples

In this experiment, 30 healthy Qinchuan bulls of similar age (15 ± 3 months) and similar initial weight (280.6 ± 30.9 kg) were selected. Each bull was fed ad lib on pellets and water in a single pen according to the same diet feeding standard of the cattle farm in Ningxia province of China. Feed intake was measured daily using Osborne’s FIRE (feed intake recording equipment) System (Osborne Industries Inc., Osborne, Kansas) accordingly for a test period of 1 to 81 days; all animals had free access to water and feed during the experiment. The RFI was calculated from the initial body weight, final body weight, and daily feed intake, the calculation method as reported in our previous study [17]. The RFI in this paper was not statistically analyzed because it is based on the results of our preliminary statistical analysis [17]. It is important to note that the core of this study was genetically related microbial characteristics under the same feeding conditions (dietary feed and environment), so the effect of diet composition on RFI and microbial populations was not the focus of this study. A total of 10 extreme RFI beef cattle were classified into high RFI (HRFI) and low RFI (LRFI) groups according to RFI values. After the experiment, all cattle were humanely slaughtered at the farm the same morning after a 16-h fast, rumen fluid (named WY.H and WY.L corresponding to HRFI and LRFI groups, respectively) and fecal samples from the rectum (named FB.H and FB.L corresponding to HRFI and LRFI groups, respectively) were collected into sterile freezing tubes within 30 min after slaughter, all samples were immediately snap-frozen using liquid nitrogen, then transported to the laboratory and stored at −80 °C. All procedures of animal experiments conformed to the “Laboratory animal Guideline for ethical review of welfare (GB/T35892-2018)” regulations.

### 2.2. Sequencing Library Construction and Metagenome Sequencing

Total DNA from fecal and ruminal contents of all 10 bulls was extracted using the DNA easy powerSoil kit (Qiagen, Hilden, Germany). All DNA samples with the required concentration and integrity were randomly broken into fragments of approximately 350 bp in length using an ultrasonic fragmentation machine, followed by end ligation, the addition of poly-A tails, the addition of sequencing adapters, and polymerase chain reaction (PCR) amplification to complete the library preparation using universal primers from Illumina protocol. After the samples were quantified and diluted to 2 ng/μL (Qubit2.0, Thermo Fisher Scientific Inc., Wilmington, DE, USA), the insert size of the library was detected using Agilent 2100 (Agilent Technologies, Santa Clara, CA, USA). After the insert size was detected as expected, the effective concentration of the library was quantified to more than 3 nM by a quantitative polymerase chain reaction to ensure the quality of the library. Finally, all samples were sequenced using 150 bp paired-end sequencing on the Illumina HiSeq 4000 platform (Illumina, San Diego, CA, USA).

### 2.3. Metagenome Assembling, Gene Prediction and Abundance Analysis

First, preprocessing the raw reads obtained from the Illumina HiSeq sequencing platform, low-quality reads and poly-N sequences or those containing standard adapters were trimmed from raw data by Readfq (v8, https://github.com/cjfields/readfq, accessed on 26 October 2020) to obtain clean reads. The reads were mapped to the bovine reference genome (ARS-UCD1.3) using Bowtie 2 software (v2.2.4, accessed on 26 October 2020) [18], to remove host genome contamination (parameters set to: end-to-end, sensitive, I 200, X 400), and the clean data of all samples were assembled and analyzed using SOAPdenovo software (v2.21, http://soap.genomics.org.cn/soapdenovo.html, accessed on 26 October 2020) (parameters set to: d 1, M 3, R, u, -) to obtain scaffolds, then the assembled scaffolds were interrupted from the N connection to generate the sequence scaftigs without N. Bowtie2 software (v2.2.4) [18], was used to compare clean data from each sample with its scaftigs (parameters set to: end-to-end, sensitive, I 200, X 400), in which the unused reads from each sample were combined and mixed assembly was performed using SOAPdenovo software (v2.04, accessed on 26 October 2020,) with the same parameter settings as the single sample assembly, and then the scaftigs of the mixed assemblies were obtained. 

Filter the fragment shorter than 500 bp in all of the scaftigs for statistical analysis both generated from the single or mixed assembly. These scaftigs (>=500 bp) were subjected to gene prediction using the Meta GeneMark (v2.10, http://exon.gatech.edu/GeneMark/metagenome/Prediction, accessed on 15 December 2020) (parameters set to: gmhmmp a, d, f 3, m, AD), and the predicted open reading frames (ORFs) of >100 nt were clustered using the cluster database at high identity with tolerance (CD-HIT) (v4.5.8, http://www.bioinformatics.org/cd-hit/, accessed on 15 December 2020) to obtain a non-redundant initial gene catalog. The longest sequences were selected as representative sequences with redundancy removed (parameters set to: c 0.95, G 0, aS 0.9, g 1, d 0).

Finally, the clean data in each sample were compared to the initial gene catalog using the Bowtie2 (v2.2.4, http://bowtie-bio.sourceforge.net/bowtie2/index.shtml, accessed on 15 December 2020), filtering out genes with a number of reads <= 2 in each sample to obtain the gene catalog (Unigenes) for subsequent analysis (parameters set to: end-to-end, sensitive, I 200, and X 400), based on the number of mapped reads and the length of the gene, to obtain the abundance information of each gene in each sample.

### 2.4. Classification Annotation and Function Annotation

The unigenes were blasted to the sequences of bacteria, fungi, archaea, and viruses which were all extracted from the NR (non-redundant protein sequence) database (v2018.1.18, https://www.ncbi.nlm.nih.gov/, accessed on 18 January 2021) of the National Center for Biotechnology Information (NCBI) by DIAMOND software (v3.2, http://www.crystalimpact.com/diamond/Default.htm, accessed on 18 January 2021) (parameter settings are blastp, -e 1e-5.) [19], and the comparison results with e value <= minimum e value × 10 were selected for subsequent analysis. The lowest common ancestor (LCA) algorithm was adopted to determine the information on species annotation of the sequence. Based on the gene abundance information, abundance tables of different taxonomic classes were generated, microbial abundance was analyzed, and the results were visualized by non-metric multidimensional scaling (NMDS) (v3.5.0, R vegan package, R Core Team, Vienna, Austria). Inter-group variability was determined by analysis of similarities (ANOSIM) (R vegan package, v3.5.0), and finally, differences among inter-group species were examined using Metastats and linear discriminant analysis effect size (LEfSe) multivariate statistical analysis. A permutation test between groups was used in Metastats analysis for each taxonomy and the *P* value corrected by Benjamini and Hochberg (<0.05) was set as the cutoff.

DIAMOND software (v3.2) (parameter setting are blastp, -e 1e-5) was used to blast the unigenes to the functional database (kyoto encyclopedia of genes and genomes (KEGG) database (v2018-01-01, http://www.kegg.jp/kegg/, accessed on 18 January 2021), evolutionary genealogy of genes: non-supervised orthologous groups (eggNOG) database (v4.5, http://eggnogdb.embl.de/#/app/home, accessed on 18 January 2021), and carbohydrate-active enzymes (CAZy) database (v201801, http://www.cazy.org/, accessed on 18 January 2021), and the comparison with the highest score (one HSP >60 bits) was carried out for subsequent analysis [20]. Clustering, ANOSIM, and NMDS analyses were performed using the generated biological classification and functional abundance tables. Based on the final grouping, information generated from these analyses, further Metastats, LEfSe multivariate statistical analyses, and comparative analysis of metabolic pathways were performed to explore the composition of microbial species and functional differences between the HRFI and LRFI groups. 

Unigenes were aligned to the comprehensive antibiotic resistance database (CARD) (https://card.mcmaster.ca/, accessed on 18 January 2021) using the resistance gene identifier software(RGI) (v5.1.1, https://github.com/arpcard/rgi, accessed on 18 January 2021), with the parameter setting are blastp, e value <=10^−30^ [21]. The relative abundance of each antibiotic resistance ontology (ARO) was calculated by combining the information on the abundance distribution of unigenes, followed by displaying an abundance bar graph, abundance clustering heatmap, ARO difference analysis among groups and species attribution analysis of resistance genes (unigenes annotated to ARO).

### 2.5. Analysis of Intestinal Differential mRNA and Microbial Interactions

To investigate the relationship between host genes and gut microbes, we used the expression data of host differential genes associated with cattle RFI in the intestine transcriptome of our previous study [17] (Supplementary Table S1) to correlate with rumen microbiome abundance classification data (genus level) with a strict screening threshold (absolute logFoldchange > 3) for the expressed genes. Spearman correlations are more effective in determining gene expression and relative abundance of microbiomes compared to others (e.g., Pearson correlation); Spearman rank correlation coefficients and corresponding *P*-values were calculated using the cor. test () function, using the *P* adjust (*P*, “BH”) function in R (v3.5.0) to perform multiple comparison corrections of the *P* value to obtain the FDR value. Representative gene correlations were visualized using the R corrplot, and significantly correlated microbial and host-expressed genes were visualized using the Cytoscape (v3.9., https://cytoscape.org/, accessed on 18 January 2021).

## 3. Results

### 3.1. Experimental Animal Selection and Sequencing Quality Control

To identify microbial species and characteristics associated with RFI variation in native Chinese beef cattle, cattle with extreme RFI phenotypes were selected from our previous study [17]. The actual daily feed intake (ADFI) and RFI values were significantly higher in the HRFI group than in the LRFI group (*P* = 0.004 and *P* = 0.006, respectively), which revealed that the cattle in the LRFI group had higher feed efficiency (Supplementary Table S2), which were used in subsequent experiments. The sequencing results showed that the 20 DNA samples of ruminal and fecal contents contained an average of 13.211 Gb of raw data (12.22–14.98 Gb), with valid data ranging from 12.20 Gb to 14.97 Gb and Q20 values above 96% for all samples (Supplementary Table S3). The dilution curve for the core gene and pan gene was flat, indicating that the amount of sequencing data was adequate (Supplementary Figure S1a,b). Statistical analysis of the open reading frame (ORF) length distribution indicated that all genes were longer than 100 nt and met the requirements for subsequent analysis (Supplementary Figure S1c). By analyzing the difference in gene numbers between the HRFI and LRFI groups, the number of non-redundant genes was observed to be higher in the HRFI group than in the LRFI group (Supplementary Figure S1d), and there were 1,710,908 and 1,563,954 common genes between the rumen and feces of HRFI and LRFI groups, respectively (Supplementary Figure S1e,f).

### 3.2. The Composition of the Microbiota

**Figure 1:**
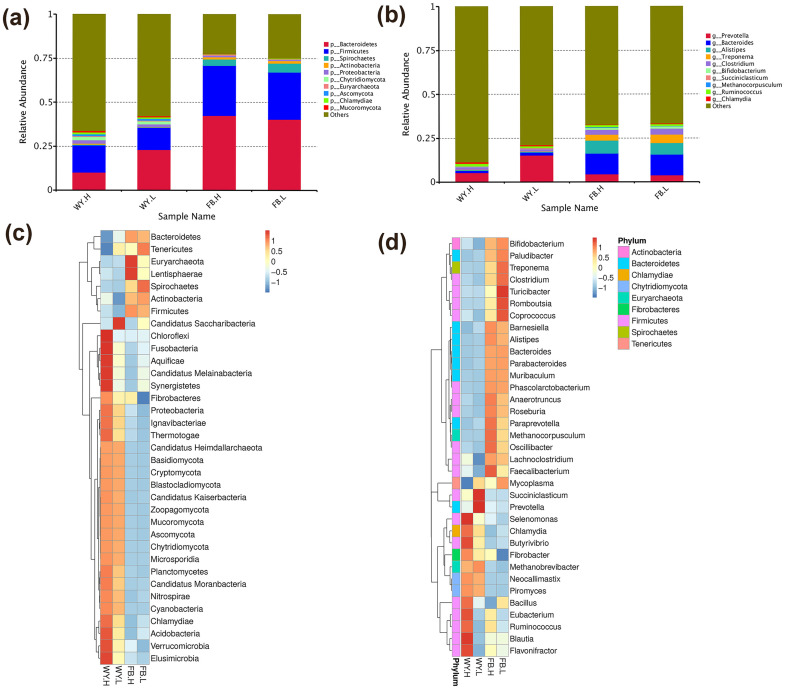
Analysis of relative species abundance at the phylum and genus levels. (**a**) Histogram of relative abundance at the phylum level; (**b**) Histogram of relative abundance at the genus level; (**c**) Heatmap of relative abundance at the phylum level; (**d**) Heatmap of relative abundance at the genus level.

Unigenes were compared to sequences from the NR (v2018-1-18) database at NCBI. Genes were classified based on taxonomy to obtain their relative abundance at each taxonomic level. The taxonomic composition of the top 10 microbiota ranked by relative abundance in the rumen and feces was analyzed and the remaining species were set to others (Figure 1). At the phylum level, *Bacteroidetes* and *Firmicutes* were the most dominant phyla in rumen and feces (Figure 1a), and both occupy more than 25% of the rumen microbiota, where *Bacteroidetes* and *Firmicutes* were 23.1% and 12.5% in the LRFI group and 10.1% and 15.5% in the HRFI group, respectively; in contrast, *Bacteroidetes* and *Firmicutes* exceeded 67% of the feces microbiota. At the genus level (Figure 1b), *Prevotella*, *Clostridium*, *Ruminococcus*, and *Bacteroides* accounted for >29% of the rumen microbiota, *Prevotella* was higher in the LRFI group (15.3%) than in the HRFI group (5.3%); the dominant genera in feces were *Prevotella*, *Bacteroides*, *Alistipes*, *Treponema*, and *Clostridium*, and their proportions were similar in both HRFI and LRFI groups. Cluster analysis of the top 35 dominant microbes of all samples, revealed that at the phylum level, 27 were enriched in the rumen and the remaining eight were enriched in the feces, with *Bacteroidetes* and *Firmicutes* significantly enriched (*P* < 0.05) in the feces (Figure 1c). At the genus level, *Prevotella* and *Bacteroides* were significantly enriched (*P* < 0.05) in the rumen and feces. In addition, some fungi and archaea were also present in these first 35 dominant genera, where the archaea *Methanocorpusculum* were enriched in the feces and the archaea *Methanobrevibacter* were enriched in the rumen; the fungi *Neocallimastix* and *Piromyces* were enriched in the rumen of the HRFI group (Figure 1d). The above results indicated that there was a difference in the distribution of microbial species in the rumen and feces of two divergent RFI groups.

To understand the differences in the microbial composition of rumen and feces in different RFI groups, downscaling was performed using NMDS to calculate the Bray−Curtis distance between pairs of samples at the species level (Supplementary Figure S2a). The results revealed clear clustering of the rumen and fecal samples, respectively, indicating differences in the rumen and feces microbiota. ANOSIM results showed that the feces (ANOSIM, R = −0.128, *P* = 0.933) of the HRFI and LRFI groups had a similar microbiota composition whereas rumen (ANOSIM, R = 0.048, *P* = 0.271) of the HRFI and LRFI groups exhibited differences in microbiota composition (Supplementary Figure S2b,c). The LEfSe (linear discriminant analysis (LDA) effect size) analysis was used to identify bacteria that were different in abundance between the high and low RFI groups in the rumen and feces. In the rumen microbiota, only two differential species were identified in the LRFI group and both were from *Prevotella* (Supplementary Figure S3a), but there weren’t any microbial species detected in the rumen HRFI group; meanwhile, in the fecal microbiota, two *Turicibacter* species in the LRFI group and *Prevotellaceae* and *Prevotella* in the HRFI group were specifically identified (Supplementary Figure S3b); we speculate that these microbial species might be potential biomarkers for different RFI groups.

### 3.3. Function Analysis

**Figure 2:**
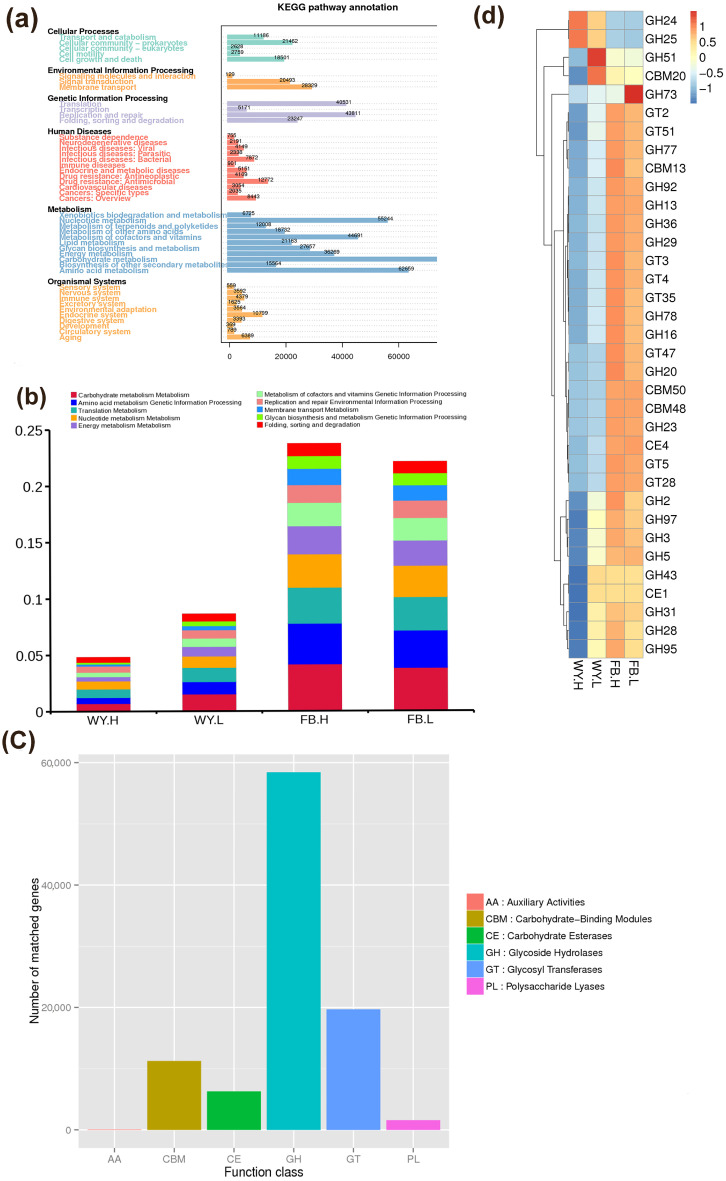
Relative abundance of microbial functions in beef cattle with different RFIs. (**a**) Statistical chart of the number of KEGG annotated genes; (**b**) The relative abundance of top ten KEGG pathways in each group; (**c**) The abundance of six types of CAZy in each group; (**d**) Heatmap of relative abundance of CAZy family clusters in each group.

KEGG has been used broadly to analyze metagenomics data [22], for understanding unigene function and the roles of microbes [23]. The unigenes of the rumen and fecal samples were aligned to the KEGG database (Figure 2a), revealing that the metabolic pathway was enriched for the highest number of unigenes. The top 10 pathways with the highest relative abundance were further compared (Figure 2b); the enrichment level of carbohydrate metabolism, translation, nucleotide metabolism, amino acid metabolism, signaling, replication and repair, energy metabolism, cofactor, vitamin metabolism, and transport and catabolic pathways were higher in the LRFI group compared with the HRFI group in the rumen. Analysis using LEfSe showed the KEGG orthology (KO), such as uridine kinase (K00876) and glutathione peroxidase (K00432), was more abundant in the LRFI group whereas glucose-1-phosphate adenosyltransferase (K00975), dCTP deaminase (K01494), DNA replication protein (K02315), and peptidoglycan DL-peptide endopeptidase CwlO (K21471) were more abundant in the HRFI group (Supplementary Figure S4a). The NMDS analysis indicated no significant difference between the HRFI and LRFI groups in the feces (Supplementary Figure S4b). They displayed similar functional annotation results for both groups in feces, which might have been owing to the similar microbiota composition of the feces (ANOSIM, R = −0.128, *P* = 0.933) in them.

CAZy is used to understand and compare the ability of an organism or a community to assemble and break down complex carbohydrates [24]. In this study, CAZy functional annotation analysis revealed that glycoside hydrolases (GHs) and glycosyltransferases (GTs) were dominant in all samples (Figure 2c). At the enzyme family level, GH51 and CBM20 were the most abundant in the rumen of the LRFI group, as well as GH24 and GH25 in the rumen of the HRFI group (Figure 2d). LEfSe analysis showed that in the rumen, the relative abundances of GT30, CE19, GT19, GH141, GH139, and PL17 were higher in the LRFI group whereas those of GH70, GT31, GBM61, GH104, CBM25, CBM34, GT8, and GH18 were higher in the HRFI group (Supplementary Figure S4c). In the feces, the abundances of GH19, GH26, GH130, and GH108 were higher in the LRFI group and that of CE7 was higher in the HRFI group (Supplementary Figure S4d).

**Figure 3:**
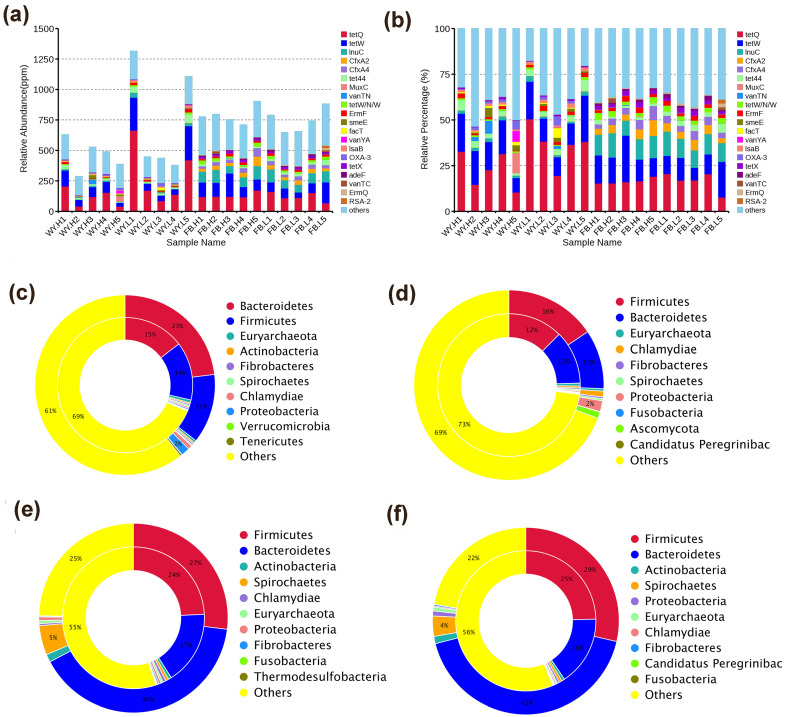
Analysis of antibiotic resistance genes; the inner circle shows the species distribution of AROs, and the outer circle shows the species distribution of all sample genes in the group. (**a**) Relative abundance of ARO in individual samples for all genes in ppm, owing to the magnification of the raw data of relative abundance by 106 times; (**b**) Relative abundance of the top 20 AROs in all AROs; (**c**) Source of LRFI rumen resistance genes; (**d**) Source of HRFI rumen resistance genes; (**e**) Source of LRFI fecal resistance genes; (**f**) Source of HRFI fecal resistance genes.

Host characteristics, environment, dietary composition, and the use of feed additives can cause the gut microbiota to display different characteristics [25]; the animal gut microbiota is a reservoir of antibiotic resistance genes (ARGs) [26]. In this study, CARD was used to identify ARGs and AROs in the rumen and fecal microbiota of beef cattle at different RFI levels. The identified AROs were further classified according to their drug resistance and sequence similarity. Downstream analysis was performed based on the abundance table of AROs (Figure 3a), and the major AROs observed in the LRFI and HRFI groups were tetracycline, lincomycin antibiotic-streptomycin antibiotic-thymine antibiotic, and macrolide antibiotic-lincomycin antibiotic-streptomycin antibiotic. In contrast, tetracycline was observed in abundance in almost all samples, and the resistance genes were mainly tetQ, tetW, InuC, tet44, and CfxA2 (Figure 3b). There were 332 and 329 common genes between the rumen and feces of the HRFI and LRFI groups, respectively (Supplementary Figure S5a,b). An analysis of the sources of resistance genes demonstrated that the AROs in the rumen bacteria of the HRFI group of Qinchuan cattle mainly belonged to *Firmicutes* and *Bacteroidetes* (Figure 3c,d), the AROs in the HRFI group were mainly from *Firmicutes* and *Bacteroidetes*, *Ascomycota*, *Fusobacteria*, *Candidatus Peregrinibacteria*, and those in the LRFI group were mainly from *Bacteroidetes* and *Firmicutes*, *Actinobacteria*, *Verrucomicrobia*, and *Tenericutes*. In the feces (Figure 3e,f), antibiotic-resistant organisms within the LRFI and HRFI group were mainly derived from *Firmicutes* and *Bacteroidetes*, the percentage of each phylum is listed in Supplementary Table S4.

### 3.4. RFI-Related Host-Gene Expression and Rumen Microbial Association Analysis

**Figure 4:**
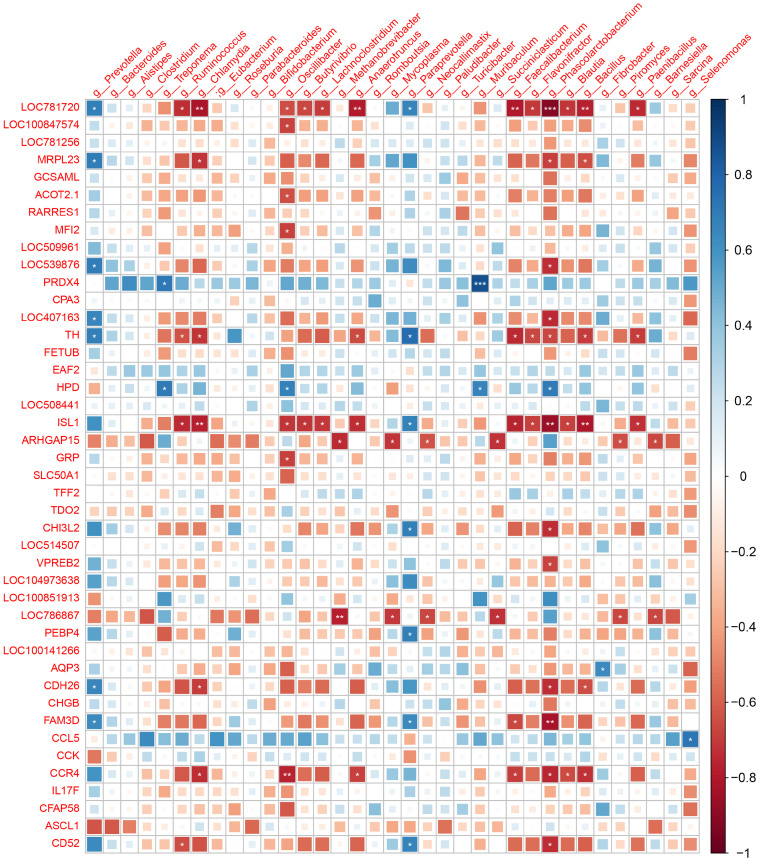
Correlation analysis of hosts’ differentially expressed genes and microbiota. The color and size of the square indicate the degree of correlation; the asterisk indicates the significance of the correlation (** indicates *P* < 0.01, * indicates *P* < 0.05); blue indicates positive correlation; red indicates negative correlation.

**Figure 5:**
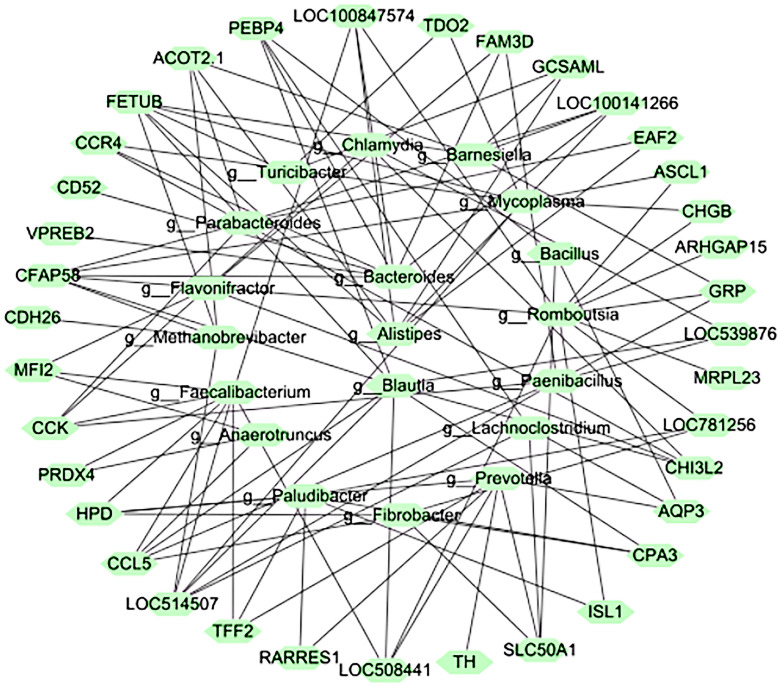
Network diagram of microbe-gene relationships with significant correlation.

The animal microbiome is a powerful modifier of host biology, influencing many phenotypic characteristics of species. In addition to nutritional and metabolic coupling, the microbiota were considered to be modulators of complex animal phenotypes (including behavior) [27,28]. We associated microbe abundance with host-expressed genes from the same individual [17]. The association between differentially expressed genes and RFI in the duodenum was determined using Spearman correlations. A significant correlation (*P* < 0.01) was observed between differentially expressed genes and microbiota, where the magnitude of the correlation (Spearman) ranged from −0.77 to 0.79, and the abundance of this correlation was significantly different between the HRFI and LRFI groups. Correlations between genus-level microbiota and host differentially expressed genes (Figure 4), and network plots with significant correlations revealed that *Prevotella* was associated with LOC781720, MRPL23, LOC539876, LOC407163, CDH26, FAM3D, and tyrosine hydroxylase (TH) (Figure 5); *Paraprevotella* exhibited significant positive correlations with LOC781720, TH, ISL1, CHI3L2, PEBP4, FAM3D, and CD52, as well as *Treponema*, *Oscillibacter*, *Muribaculum* and *Phascolarctobacterium* displayed significant positive correlations with 4-hydroxyphenylpyruvate dioxygenase (HPD) genes.

Additionally, many microbes, such as *Ruminococcus, Chlamydia, Oscillibacter, Butyrivibrio, Lachnoclostridium, Methanobrevibacter, Anaerotruncus, Faecalibacterium Flavonifractor, Phascolarctobacterium, Blautia, Bacillus*, and *Paenibacillus,* were observed to be positively associated with differentially expressed genes such as LOC781720, MRPL23, TH, ISL1, ARHGA15, and LOC786867. However, many microbes, such as *Ruminococcus, Chlamydia, Oscillibacter, Butyrivibrio, Lachnoclostridium, Methanobrevibacter, Anaerotruncus, Faecalibacterium Flavonifractor, Phascolarctobacterium, Blautia, Bacillus*, and *Paenibacillus,* were observed to be negatively associated with differentially expressed genes such as LOC781720, MRPL23, TH, ISL1, ARHGA15, LOC786867, CDH26, and carbon catabolite repression 4 (CCR4). The results mentioned above suggest a significant correlation between host gene expression and its gut microbes; however, further experiments are needed to verify how microbial composition diversity and structure are involved in regulating or influencing host gene expression.

## 4. Discussion

Metagenomics is an analysis of the entire genetic material of microbiota in the living environment and is an important way to understand the taxonomic and functional characterization of intestinal microbiota [29]. General gut microbes of animals have been called the second genome of individuals, and there is growing evidence that microbial components are correlated with host genetics [30]. The bovine gastrointestinal tract contains a diverse microbial ecosystem, microbiota from the rumen or gut are involved in regulating host metabolism and health by providing energy through fermentation of undigested meals, and even small changes in microbiota can drastically impact livestock nutrition absorption and productivity [31]. Numerous studies have reported that microbiota present in the gastrointestinal tract was closely related to cattle lactation, fat deposition, feed efficiency, and disease [32,33,34]. Although metagenomics has been used in various animals, such as humans, pigs, and mice, few studies using it to investigate cattle with different RFI phenotypes and correlate the microbiota abundance with host gene expression have been reported.

Indeed, previous studies have identified links between the rumen microbiome and genetic variation in farm-animal feed-efficiency phenotypes [35,36]. In this study, the HRFI group consumed 11.97% more feed than the LRFI group (Supplementary Table S2), and actual daily feed intake showed significant differences between the two groups (*P* = 0.04); this means lower feed efficiency for beef cattle in the HRFI group [37]. Metagenome analysis of microbiota from both HRFI and LRFI groups revealed that, at the phylum level, *Bacteroidetes* and *Firmicutes* were the most abundant microbiota in the rumen and feces, consistent with previous studies [38,39,40], and the abundance of *Bacteroidetes* in the rumen was higher in the LRFI group (23.1%) than in the HRFI group (10.1%). Studies have shown that *Bacteroidete*, *Firmicutes* and *Prevotella* can decompose polysaccharides that cannot be digested by the host in the intestinal tract, promoting the digestion and absorption of nutrients which provide energy for the host [41,42,43]; these bacteria may have an impact on host feed efficiency by affecting the volatile fatty acid concentration [44]. The main products of *Bacteroidete* are phosphobutyryltransferase and butyrate kinase [45], which promote the fermentation of resistant starch, indigestible oligosaccharides, and their derivatives in the rumen for energy supply, which is conducive to improving feed utilization in ruminants [46], thereby reducing feed costs. It was reported that a decrease in the number of *Bacteroidetes* in the digestive microbiota of mice causes an increase in lipids and the ability to alter fat deposition in different tissues [47]. The results of this study displayed that at the genus level, *Prevotella*, *Bacteroides*, and *Clostridium* were the dominant genera in the rumen and feces of each group [48]. In this study, the abundance of *Prevotella* was higher in the LRFI group (15.3%) than in the HRFI group (5.3%). LEfSe analysis demonstrated that *Prevotella* sp. *tc2_28* and unclassified *Prevotellacea* might be potential biomarkers for the LRFI group in the rumen, because *Prevotella*-rich microbiota can improve growth performance and play a remarkable role in the regulation of RFI in beef cattle [49]. *Prevotella*, as one of the core rumen microbes [50], is widely present in the rumen [51]. The proportion of *Prevotella* is positively correlated with traits including feed intake [52], feed efficiency [53], and body weight gain [54] in animals. *Prevotella* is considered to be a polysaccharide-degrading bacteria [55], *Prevotella* uses other cellulolytic bacteria to degrade cellulose into acetic acid and small amounts of isobutyric acid, isovaleric acid, and lactic acid to provide a source of energy for the host [6]. Additionally, *Prevotella* can significantly promote carbohydrate and nitrogen metabolism in ruminants [56,57], and synthesize new polypeptides [58], and has been commonly observed in numerous species, including humans [59], primates [60], mice [61], ruminants [62], and poultry [63]. Reports showed that gut microbiome composition differences impact feed efficiency in pig [13], sheep [64], and cattle [12]. Combined with our results, the above results suggest that there is a complex link between microbiota (types or abundances) and host phenotype (feed efficiency) in cattle and demonstrate that the effect of microbiota on host feed efficiency has far more impact than we think.

The ruminal microbial function may reflect the potential mechanisms by which microbes influence individual phenotypes. Functional enrichment of rumen microbes showed an increased relative abundance of carbohydrate metabolism, energy metabolism, and transport and catabolic pathways in the LRFI group relative to the HRFI group, suggesting greater utilization of feed by rumen microbes in the LRFI group. KEGG orthology related to the metabolism of proteins, nucleotides, cofactors, vitamins, and monosaccharides or energy transport was more abundant in pigs with high feed efficiency, consistent with the results of our study [65]. Furthermore, KEGG orthology associated with nitrogen metabolism, amino acid metabolism, and transport systems was positively correlated with pig feed efficiency [66]. One study on the functional enrichment of rumen microbiota in cows with different feed conversion rates (FCR) found that acid metabolism, carbohydrate metabolism, energy metabolism, and vitamin metabolic pathways were more abundant in cows with high FCR (LRFI) than those with low FCR (HRFI) [67]; this indicates interactions among different microbiota affecting RFI and energy collection in dietary feeds. Analysis of rumen and fecal resistant gene sources revealed that resistant genes were mainly derived from *Firmicutes* and *Bacteroidetes*, genes encoding broad-spectrum carbohydrate-activating enzymes, particularly GHs and GTs, which were reported to be enriched in the *Bacteroidetes* genome [68], which is consistent with the results of our study. This indicates that microbes such as *Firmicutes* and *Bacteroidetes* can promote feed utilization and improve production performance in beef cattle.

As part of the small intestine, the duodenum contains many intestinal microbiotas that may affect the health and feed efficiency of beef cattle. To understand the impact of the interaction between host genes and microbiota diversity on RFI, this study demonstrated the correlation of differentially expressed genes in the duodenal epithelium with rumen microbiota by integrating rumen microbiome and host duodenal gene expression profiles. Previous studies have revealed that the bacterial profiles in the rumen of efficient beef cattle differed from those of inefficient ones, and the abundances of bacterial genera such as *Butyrivibrio, Lactobacillus, Prevotella, Ruminococcus*, and *Succinivibrio* were associated with feed efficiency traits, including RFI, dry matter intake in beef steers [39,69,70] and heifers [71]; this is similar to the results of our study. In our study, *Succiniclasticum* showed a significant positive correlation with the ARHGAP15 gene (*P* < 0.05), and ARHGAP15 negatively regulates RFI (Supplementary Table S1). The results of a previous study displayed that *Succiniclasticum* genera were significantly more abundant in low FCR animals, corroborating the results of our experiment [72]. There is evidence that *Succiniclasticum* is positively correlated to average daily weight gain [73]. It is beneficial by improving host feed efficiency, indicating that *Succiniclasticum* might interact with the ARHGAP15 gene to regulate the RFI of beef cattle. In this study, FAM3D exhibited a positive correlation with *Prevotella* and *Paraprevotella*, and the abundance of *Prevotella* in the rumen was significantly higher in the LRFI group than in the HRFI group. FAM3D is mainly involved in pathways related to digestion, substance metabolism, immunity, and glucose-energy metabolism in animals. FAM3D is highly expressed in the human placenta and gastrointestinal tract, and the expression level of FAM3D is reduced in the fasting state and increased after eating [74]. This indicates that FAM3D may be an important gene that influences the variability of RFI in farm animals, but how FAM3D interacts with *Prevotella* and *Paraprevotella* to participate in the regulation of energy metabolism is not clear. Highly intertwined interactions between host transcriptomes and enteric microbiota are likely to modulate animals’ complex traits; it has been reported that 10% of the intestinal transcriptome of adult mice is regulated by intestinal microbiota [75]. Research has shown that gene transcription in low and high FCR broilers was significantly associated with the abundance of specific microbial taxa, for example, heat shock protein HSP90AA1 transcription was positively correlated with the abundance of *Clostridium, Weissella,* and *Bacillus* was negatively correlated with the abundance of the potentially pathogenic *Gallibacterium* and *Veillonella*; furthermore, the abundances of *Gallibacterium* and *Veillonella*, as well as *Faecalibacterium*, were negatively correlated with the transcription of the anti-viral protein, G3BP [76]. These results suggest a potential association between feed efficiency and rumen microbiome and that microbiota may participate in the regulation of complex traits through certain metabolic processes in the gut of livestock.

An increasing number of studies have shown that microbiota plays a crucial role in the study of human diseases [77], fat deposition [78], body weight [79], reproduction [80], obesity [81], and feed intake [82]. In this study, we found that rumen and fecal microbiota from different RFI groups in beef cattle were characterized differently and closely associated with host gene expression, which provides an important basis for subsequent studies on the involvement of microbiota in host trait formation through epigenetic regulation; however, functional studies of key microbiota still require extensive in vivo and in vitro experiments to be confirmed.

## 5. Conclusions

The ruminal and fecal microbiota metagenome features associated with RFI in Chinese Qinchuan cattle were studied. The highest abundances of *Prevotella*, *Bacteroides*, and *Clostridium* in the rumen and feces of Qinchuan cattle were identified. The microbial species and abundance in the feces were similar and microbiota in the rumen LRFI group had more utilization of polysaccharides and energy metabolism. In addition, differentially expressed genes associated with RFI in intestinal epithelial tissues were significantly correlated with microbiota. These findings clarify the differences in the rumen and fecal microbiota composition and structure of Chinese indigenous beef at different RFI levels, expand the knowledge of microbes on feed intake, and provide a basis for regulating the feeding behavior and feed efficiency of beef cattle.

## Data Availability

The dataset generated from this study is available at the NCBI Gene Expression Omnibus database (http://www.ncbi.nlm.nih.gov/geo/, accessed on 12 May 2021), which can be accessed through GEO series login number GSE76501.

## Supplementary Materials

The following supporting information can be downloaded at: https://www.mdpi.com/article/10.3390/microorganisms11020358/s1, Figure S1: Gene abundance statistics. (a) Core genome sparsity curve; (b) Pan-genome sparsity curve; (c) Gene length distribution plot; (d) Box plot showing the difference in gene numbering between two groups; (e) Number of shared and unique genes between two groups; (f) Number of shared and unique genes between two groups; Figure S2: Non-metric multidimensional scaling (NMDS) results. (a) andANOSIM analysis based on gate-level (b,c). Figure S3: Distribution of linear discriminant analysis (LDA) values and evolutionary branching of divergent species. (**a**) WY.H VS WY.L; (**b**) FB.H VS FB.L. Figure S4: Functional analysis of rumen and fecal microbiota. (a) KEGG analysis of rumen microbiota; (b) NMDS analysis of feces microbiota; (c) KEGG analysis of rumen microbiota (d) KEGG analysis of feces microbiota; Distribution of linear discriminant analysis (LDA) values for different functions: (c) WY.H VS WY.L; (d) FB.H VS FB. Figure S5: Venn diagram of resistance gene box among the two groups. (a) WY.H VS WY.L; (b) FB.H VS FB.L. Table S1: 43 differentially expressed genes in intestinal transcriptome at different RFIs; Table S2: Significance analysis of production performance of Qinchuan cattle with different RFI (Mean ± SD); Table S3: Raw data filtering statistics; Table S4: The percent for all phyla in rumen and feces samples.
