# Supplementary material for: Rumen and Fecal Microbiota Characteristics of Qinchuan Cattle with Divergent Residual Feed Intake
Source: Microorganisms. 2023 Jan 31;11(2):358. doi: 10.3390/microorganisms11020358 (PMC9964965; doi:10.3390/microorganisms11020358)
Supplement: Supplementary file 1 [file microorganisms-11-00358-s001.zip › microorganisms-2092032-supplementary.pdf]

microorganisms

MDPI

Article

Rumen and Fecal Microbiota Characteristics of Qinchuan Cattle with Divergent Residual Feed Intake

Xiaonan Zhou †, Ying Ma †, Chaoyun Yang, Zhiyan Zhao, Yanling Ding, Yanfeng Zhang, Pengfei Wang, Lei Zhao, Chenglong Li, Zonghua Su, Xiaowei Wang, Wenxun Ming, Ling Zeng and Xiaolong Kang *

Key Laboratory of Ruminant Molecular and Cellular Breeding, School of Agriculture, Ningxia University, Yinchuan, China
* Correspondence: kangxl9527@126.com
†These authors contributed equally to this work.

Supplementary Figures

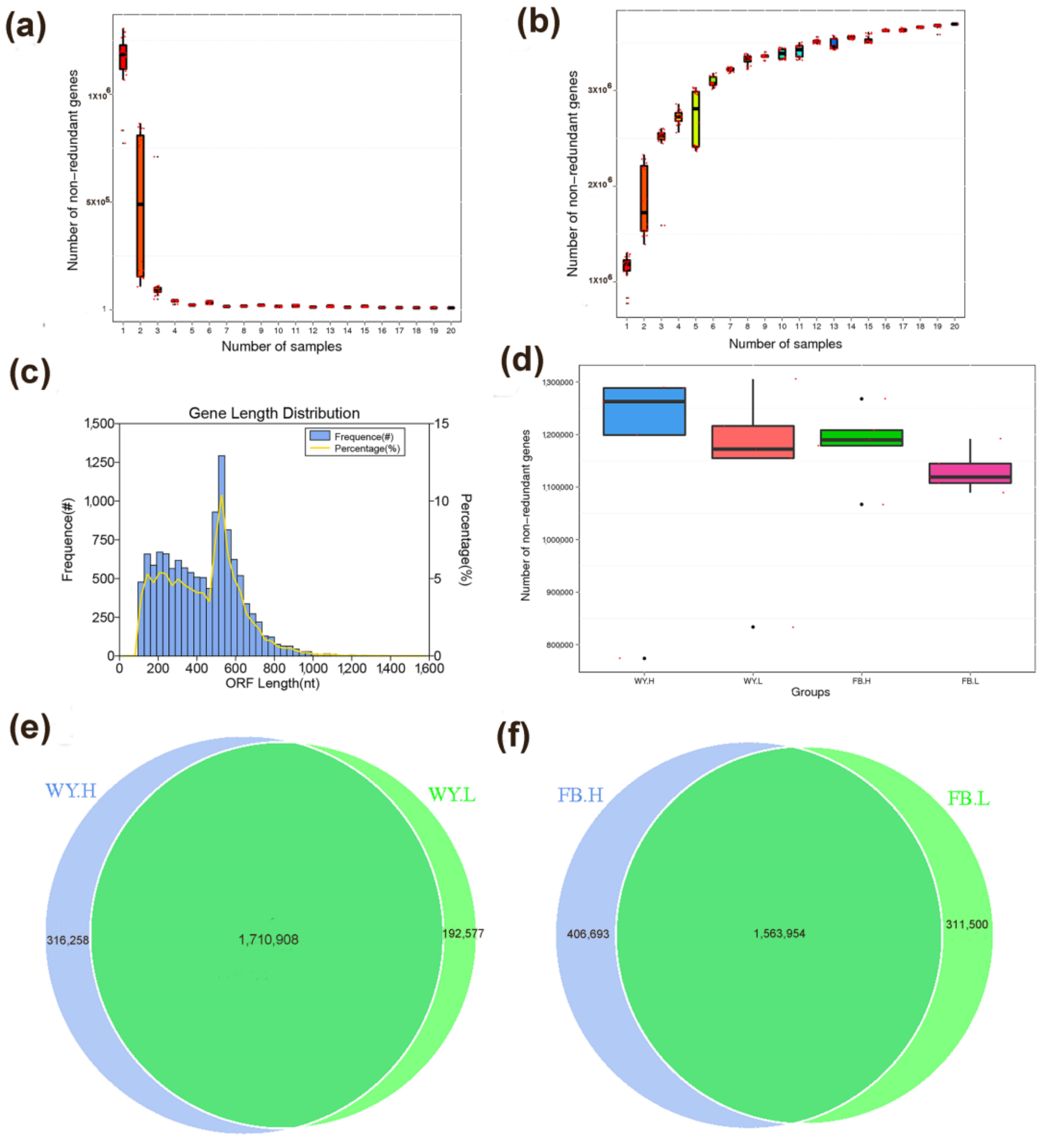

Figure S1. Gene abundance statistics. (**a**) Core genome sparsity curve; (**b**) Pan-genome sparsity curve; (**c**) Gene length distribution plot; (**d**) Box plot showing the difference in gene numbering

between two groups; (**e**) Number of shared and unique genes between two groups; (**f**) Number of shared and unique genes between two groups.

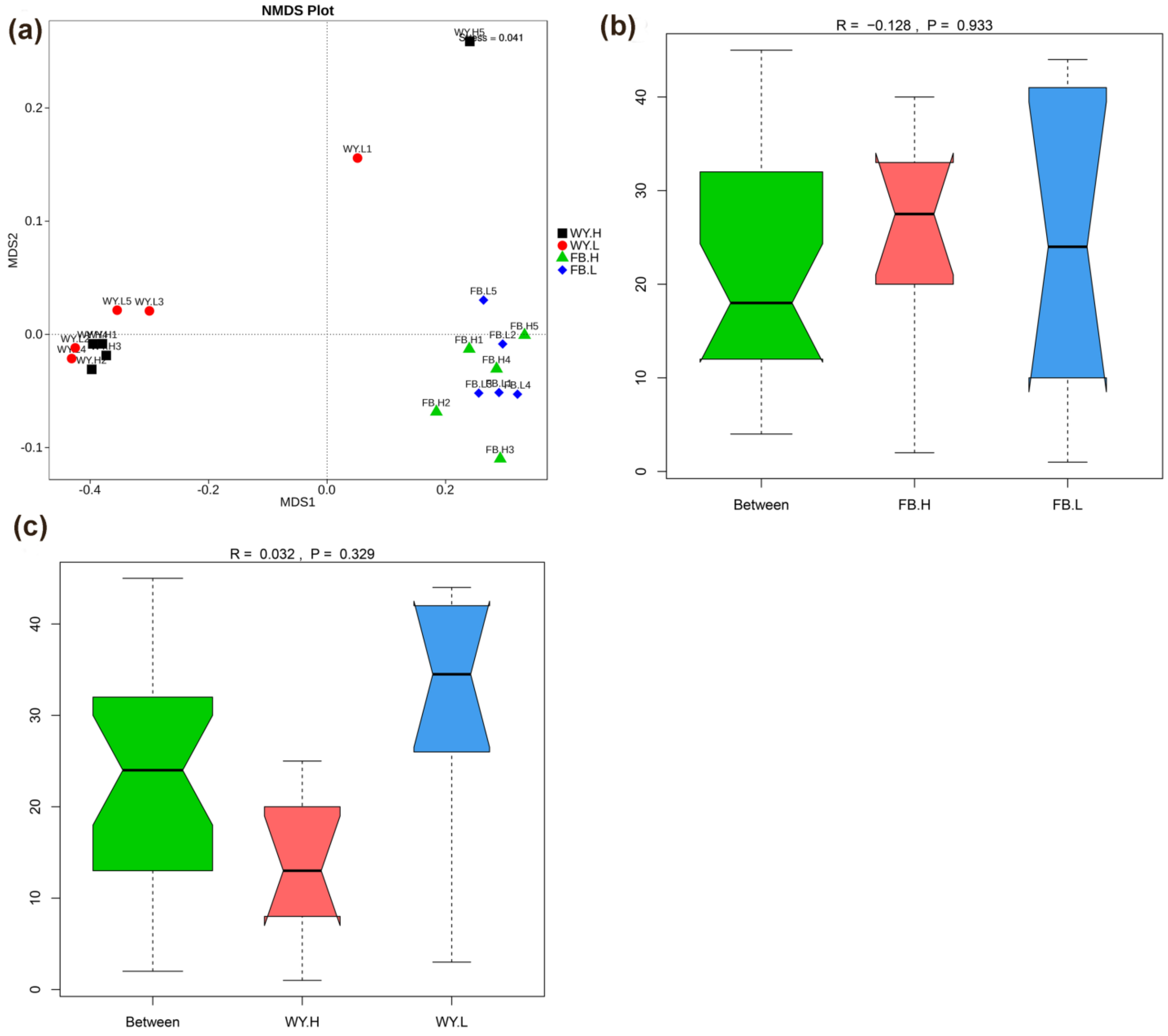

Figure S2. Non-metric multidimensional scaling (NMDS) results. (**a**) andANOSIM analysis based on gate-level (**b, c**).

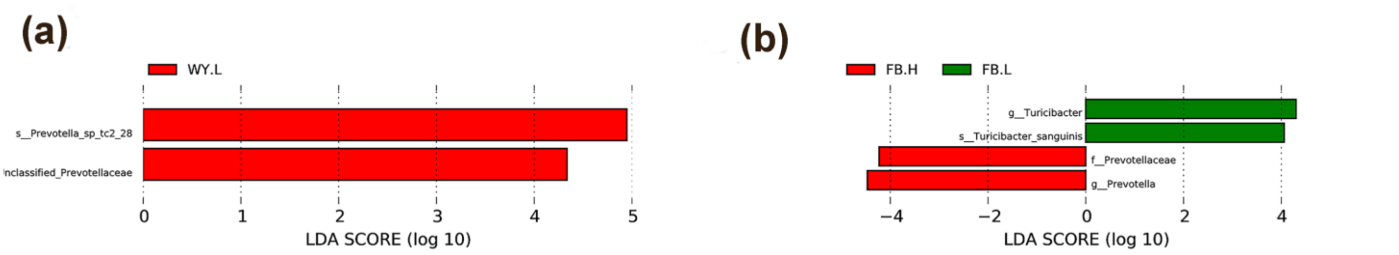

Figure S3. Distribution of linear discriminant analysis (LDA) values and evolutionary branching of divergent species. (**a**)WY.H VS WY.L; (**b**)FB.H VS FB.L.

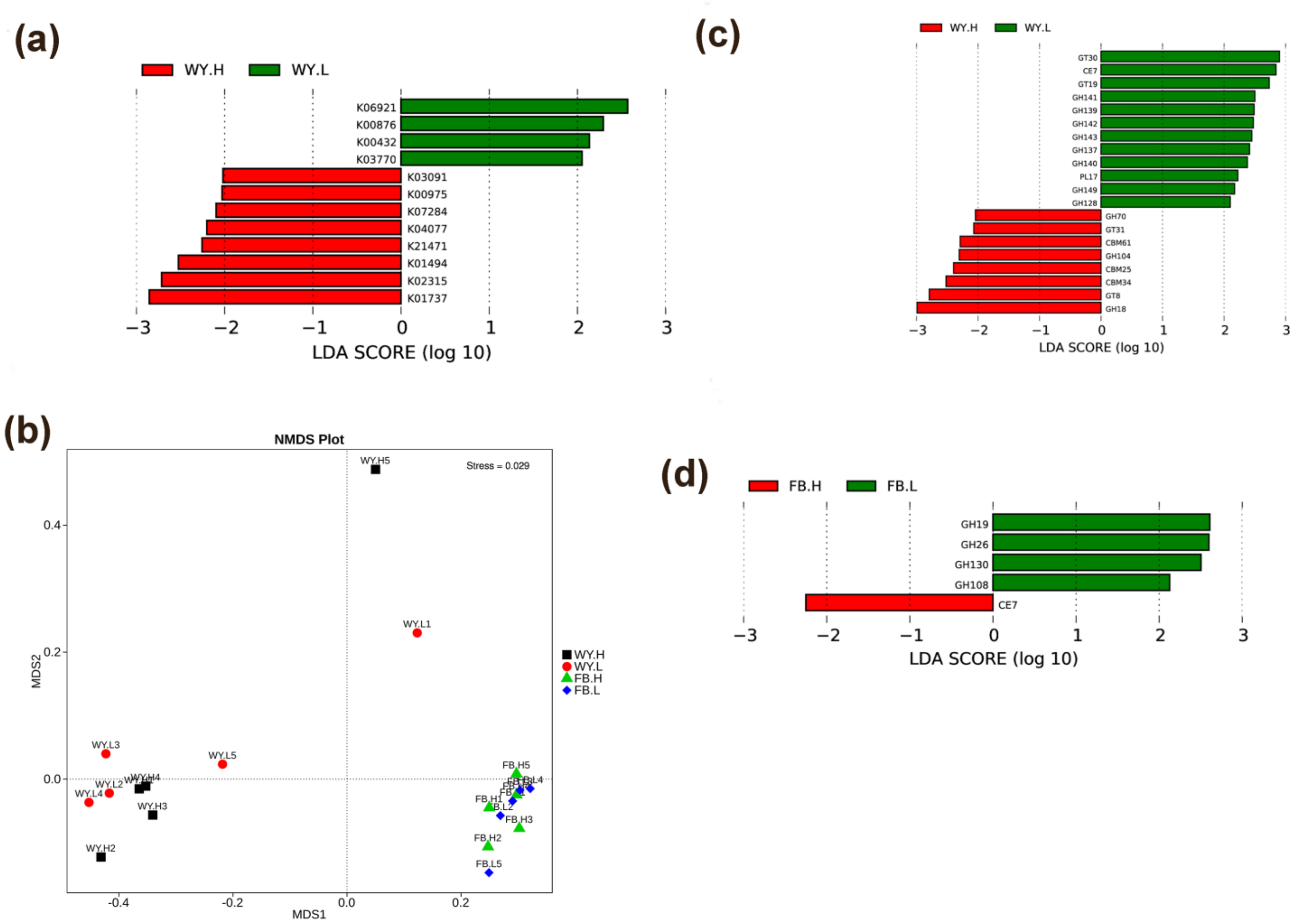

Figure S4. Functional analysis of rumen and fecal microbiota. (**a**) KEGG analysis of rumen microbiota; (**b**) NMDS analysis of feces microbiota; (**c**) KEGG analysis of rumen microbiota (**d**) KEGG analysis of feces microbiota; Distribution of linear discriminant analysis (LDA) values for different functions: (**c**) WY.H VS WY.L; (**d**) FB.H VS FB.L.

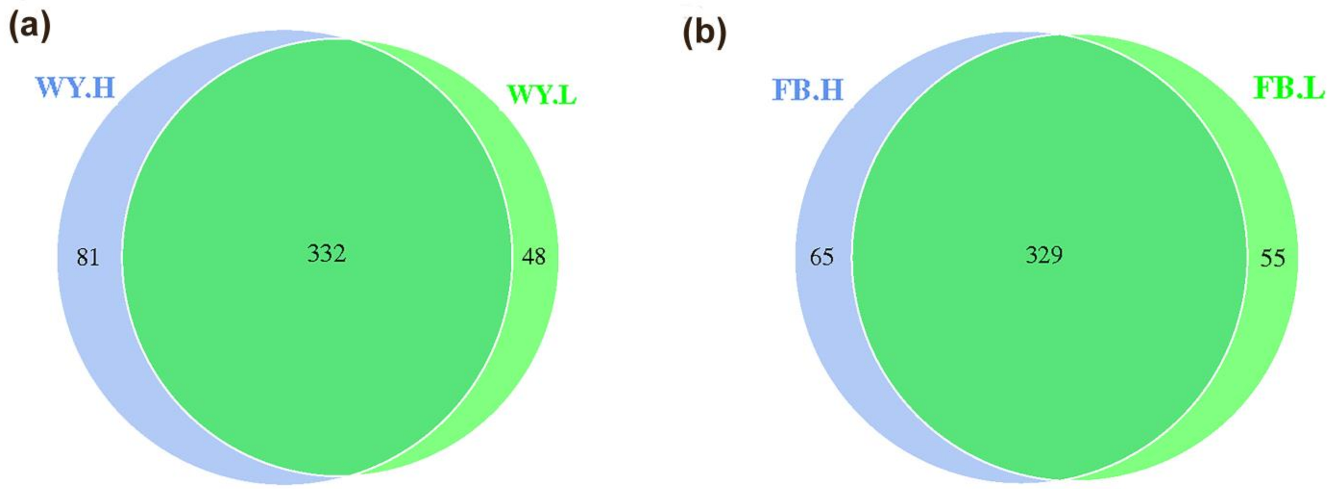

Figure S5. Venn diagram of resistance gene box among the two groups. (**a**) WY.H VS WY.L; (**b**) FB.H VS FB.L.

Supplementary Tables

Table S1. 43 differentially expressed genes in intestinal transcriptome at different RFIs.

Gene Name	log2（Foldchange）	*P*-Value	FDR	Style
LOC781720	3.0956	0.0000	0.0000	up
LOC100847574	4.4684	0.0000	0.0000	up
LOC781256	−5.1688	0.0000	0.0000	down
MRPL23	−0.9338	0.0000	0.0000	down
GCSAML	2.3837	0.0000	0.0000	up
ACOT2.1	−3.4098	0.0000	0.0001	down
RARRES1	−1.2230	0.0000	0.0002	down
MFI2	−1.5617	0.0000	0.0003	down
LOC509961	−1.1876	0.0000	0.0008	down
LOC539876	−1.2169	0.0000	0.0014	down
PRDX4	−0.6009	0.0000	0.0014	down
CPA3	1.8477	0.0000	0.0017	up
LOC407163	−6.6653	0.0000	0.0017	down
TH	1.6242	0.0000	0.0021	up
FETUB	4.9362	0.0000	0.0024	up
EAF2	−0.9485	0.0000	0.0032	down
HPD	−2.1522	0.0000	0.0044	down
LOC508441	1.7676	0.0000	0.0046	up
ISL1	1.0655	0.0000	0.0046	up
ARHGAP15	−0.7072	0.0000	0.0052	down
GRP	1.6598	0.0000	0.0073	up
SLC50A1	−0.7412	0.0000	0.0085	down
TFF2	−0.7987	0.0000	0.0086	down
CHI3L2	−3.4622	0.0000	0.0098	up
TDO2	1.4534	0.0000	0.0098	down
LOC514507	4.7850	0.0000	0.0111	up
VPREB2	−1.9922	0.0000	0.0112	down
LOC104973638	−7.0704	0.0000	0.0112	down
LOC100851913	−2.6272	0.0000	0.0114	down
LOC786867	4.3015	0.0000	0.0143	up
PEBP4	−0.9701	0.0000	0.0170	down
LOC100141266	−1.3293	0.0000	0.0191	down
AQP3	−0.8545	0.0000	0.0206	down
CDH26	3.1660	0.0000	0.0211	up
CHGB	1.3096	0.0000	0.0261	up
FAM3D	−0.5922	0.0001	0.0266	down
CCL5	−0.8184	0.0001	0.0266	down
CCK	1.3095	0.0001	0.0297	up
CCR4	−1.6659	0.0001	0.0314	down
IL17F	−5.5405	0.0001	0.0351	down
CFAP58	−2.5937	0.0001	0.0351	down
ASCL1	2.1796	0.0001	0.0426	up
CD52	−0.8999	0.0001	0.0447	down

Table S2. Significance analysis of production performance of Qinchuan cattle with different RFI (Mean ± SD).

	HRFI Group	LRFI Group	*P*-Value
Initial body weight(kg)	273.50 ± 20.28	285.75 ± 22.32	0.448
Final body weight (kg)	412.50 ± 35.10	419.25 ± 28.72	0.776
Average daily gain (kg/d)	1.187 ± 0.06	1.21 ± 0.047	0.450
Actual daily feed intake (kg)	11.86 ± 0.08	10.44 ± 0.23	0.004
RFI (kg)	0.55 ± 0.044	−0.84 ± 0.13	0.006

SD standard deviation.

Table S3. Raw data filtering statistics.

Sample Name	Raw Data	Clean Data	Q20 Content (%)	Q30 Content (%)	GC Content (%)
WY.H1	13,480.62	13,428.48	97.20	91.98	34.19
WY.H2	13,006.87	12,962.35	97.07	91.72	29.27
WY.H3	13,754.91	13,711.80	97.28	92.19	32.36
WY.H4	13,612.34	13,572.89	97.27	92.21	33.16
WY.H5	14,975.90	14,964.36	96.52	90.67	42.92
WY.L1	13,129.56	13,122.49	97.49	92.80	47.48
WY.L2	13,135.14	13,113.61	97.35	92.22	30.36
WY.L3	12,668.74	12,631.11	97.25	92.17	35.38
WY.L4	13,050.74	13,032.51	96.88	91.19	29.32
WY.L5	13,020.91	12,986.51	97.30	92.31	39.72
FB.H1	12,664.17	12,641.64	97.56	92.97	45.66
FB.H2	12,812.16	12,799.09	97.53	92.79	45.33
FB.H3	12,228.62	12,209.60	97.64	93.05	43.28
FB.H4	12,793.23	12,779.56	97.38	92.46	44.27
FB.H5	13,718.24	13,691.41	97.36	92.54	44.12
FB.L1	13,365.36	13,352.09	97.08	91.79	44.42
FB.L2	13,553.91	13,543.11	97.32	92.34	43.34
FB.L3	12,897.86	12,881.15	97.30	92.29	42.97
FB.L4	12,653.89	12,644.64	97.69	93.13	43.87
FB.L5	13,702.14	13,634.43	97.69	93.17	43.12

Table S4. The percent for all phyla in rumen and feces samples.

Species Name	WY-L	WY-H	FB-L	FB-H
Bacteroidetes	15.000%	12.000%	17.000%	16.000%
Firmicutes	14.000%	12.000%	24.000%	25.000%
Euryarchaeota	0.740%	1.100%	0.440%	0.250%
Actinobacteria	0.210%		0.860%	0.860%
Fibrobacteres	0.210%	0.270%	0.200%	0.082%
Spirochaetes	0.210%	0.280%	0.940%	0.910%
Chlamydiae	0.210%	0.790%	0.920%	0.210%
Proteobacteria	0.210%	0.280%	0.400%	0.610%
Verrucomicrobia	0.100%			
Tenericutes	0.110%			
Fusobacteria		0.090%	0.130%	0.032%
Ascomycota		0.090%		

Candidatus_Peregrinibacteria		0.100%		0.046%
Thermodesulfobacteria			0.110%	
Others	69.000%	73.000%	55.000%	56.000%
